# *De novo* drug design by iterative multiobjective deep reinforcement learning with graph-based molecular quality assessment

**DOI:** 10.1093/bioinformatics/btad157

**Published:** 2023-03-24

**Authors:** Yi Fang, Xiaoyong Pan, Hong-Bin Shen

**Affiliations:** Department of Automation, Key Laboratory of System Control and Information Processing, Ministry of Education of China, Institute of Image Processing and Pattern Recognition, Shanghai Jiao Tong University, Shanghai 200240, China; Department of Automation, Key Laboratory of System Control and Information Processing, Ministry of Education of China, Institute of Image Processing and Pattern Recognition, Shanghai Jiao Tong University, Shanghai 200240, China; Department of Automation, Key Laboratory of System Control and Information Processing, Ministry of Education of China, Institute of Image Processing and Pattern Recognition, Shanghai Jiao Tong University, Shanghai 200240, China

## Abstract

**Motivation:**

Generating molecules of high quality and drug-likeness in the vast chemical space is a big challenge in the drug discovery. Most existing molecule generative methods focus on diversity and novelty of molecules, but ignoring drug potentials of the generated molecules during the generation process.

**Results:**

In this study, we present a novel *de novo* multiobjective quality assessment-based drug design approach (QADD), which integrates an iterative refinement framework with a novel graph-based molecular quality assessment model on drug potentials. QADD designs a multiobjective deep reinforcement learning pipeline to generate molecules with multiple desired properties iteratively, where a graph neural network-based model for accurate molecular quality assessment on drug potentials is introduced to guide molecule generation. Experimental results show that QADD can jointly optimize multiple molecular properties with a promising performance and the quality assessment module is capable of guiding the generated molecules with high drug potentials. Furthermore, applying QADD to generate novel molecules binding to a biological target protein DRD2 also demonstrates the algorithm’s efficacy.

**Availability and implementation:**

QADD is freely available online for academic use at https://github.com/yifang000/QADD or http://www.csbio.sjtu.edu.cn/bioinf/QADD.

## 1 Introduction

*De novo* drug design has attracted widespread attention in the past decade. In general, generating a pool of drug candidates for sequential synthesis is the first step in molecule discovery. However, many molecules with good drug potentials are not mined due to the deficient and inefficient exploration of chemical space, whose estimated magnitude reaches 10^23^–10^60^ (Kirkpatrick and Ellis 2004; Reymond 2015). When compared with the limited number of molecules deposited in databases and approved drugs, a huge number of potential drug-like molecules are still not discovered and synthesized. For example, as of December 2021, the ZINC database contains nearly 10^9^ commercially available compounds (Sterling and Irwin 2015). The ChEMBL database deposits ∼2 × 10^6^ bioactive molecules with drug-like properties (Mendez et al. 2019), of them, only 43 264 are FDA-approved drugs. The above statistics indicate that current database records only account for a small fraction of the whole chemical space, which suggests a great potential for *de novo* drug design through extensive space explorations. *De novo* drug design can greatly reduce the time and cost for drug development.

Although drugs can be discovered with different wet-lab experiments, they are generally time-consuming and costly. To accelerate the drug design process, numerous computational approaches (Olivecrona et al. 2017; Blaschke et al. 2018, 2020; De Cao and Kipf 2018; Popova et al. 2018; You et al. 2018a; Zhou et al. 2019; Grisoni et al. 2020; Jin et al. 2020) have been developed to recommend a set of potential candidates and they effectively can generate new molecules with desired properties. In recent years, deep learning-based methods have gradually been applied to molecule generation and achieved remarkable progress. These deep learning-based methods can be roughly classified into two groups.

The first group of methods learns the distribution of the training molecules by deep generative models and then generates similar molecules using the trained model. For example, an autoencoder-based method is proposed to map molecules from SMILES format to a low-dimensional latent space through an encoder, and then samples the molecules from the latent space and reconstructs the molecules using a decoder (Blaschke et al. 2018). The generative adversarial networks consisting of a generator network and a discriminator network are proposed to process molecular graphs, where the generator outputs a molecular graph from a feature vector sampled with a prior while the discriminator determines whether the molecular graph is from the training dataset or the generator (De Cao and Kipf 2018). Bidirectional Recurrent Neural Networks are applied to handle nonunivocal and nondirectional SMILES format of molecules, where two RNNs are used to collect information in the SMILES strings forward and backward, and then the collected information is combined to generate new SMILES strings (Grisoni et al. 2020).

The other group of methods aims to search molecules with desired properties in the chemical space by optimizing the objective functions, which are usually derived from a combination of multiple properties. Reinforcement learning (RL) methods are demonstrated to have good exploration and optimization ability. A graph convolutional policy network is used to guide goal-directed molecule graph generation using proximal policy optimization (PPO), which optimizes molecular properties like quantitative metrics of drug-likeness (QED; Bickerton et al. 2012), and octanol-water partition coefficient (log*P*; You et al. 2018). Similarly, a Deep Q Network (DQN) is designed for optimizing predefined molecular properties of QED and log*P* (Zhou et al. 2019) on Morgan fingerprints features (Rogers and Hahn 2010). Differently, an RL-based method is proposed to use molecular graph representation, which focuses on important molecular substructures and optimizes molecules on the properties QED (Bickerton et al. 2012), synthetic accessibility score (SAscore; Ertl and Schuffenhauer 2009) and inhibition scores against two Alzheimer-related target proteins. ReLeaSE trains a generative RL model to generate molecules and a supervised model to forecast the desired properties of the generated molecules (Popova et al. 2018). In these methods, a relatively common objective is to optimize multiple properties in parallel. To achieve this goal, these methods usually convert the multiobjective optimization into a single-objective optimization with a weighted combination, resulting in one dominant objective function.

At least two main challenges need to be addressed in designing molecules with RL methods. One challenge is how to represent the molecules in an appropriate way. Currently, there are three main types of molecular representations: SMILES strings, molecular fingerprints, and molecular graphs.

SMILES strings can be obtained through expanding the 2D structures of molecules into linear structures according to certain rules and then converting them to the corresponding ASCII strings (Weininger 1988). SMILES can reflect the structural information and atomic composition of molecules to a certain extent, but it may miss the topology information. In addition, SMILES strings are context-dependent (Liu et al., 2017), two molecules with similar SMILES strings may have different structures. It has been demonstrated that molecule generation using SMILES strings cannot ensure 100% chemical validity unless complicated constraints are introduced (Jin et al., 2018).

Molecular fingerprints (Durant et al., 2002; Rogers and Hahn, 2010) are binary sequences converted by the 2D structures of molecules according to the topological connectivity. They are widely used in measuring the similarity between molecules by calculating the Tanimoto similarity (Bajusz et al., 2015) or Euclidean distance. However, molecular fingerprints also neglect structural information of molecules, potentially resulting in a nonbijective mapping between molecules and molecular fingerprints.

**Figure 1:**
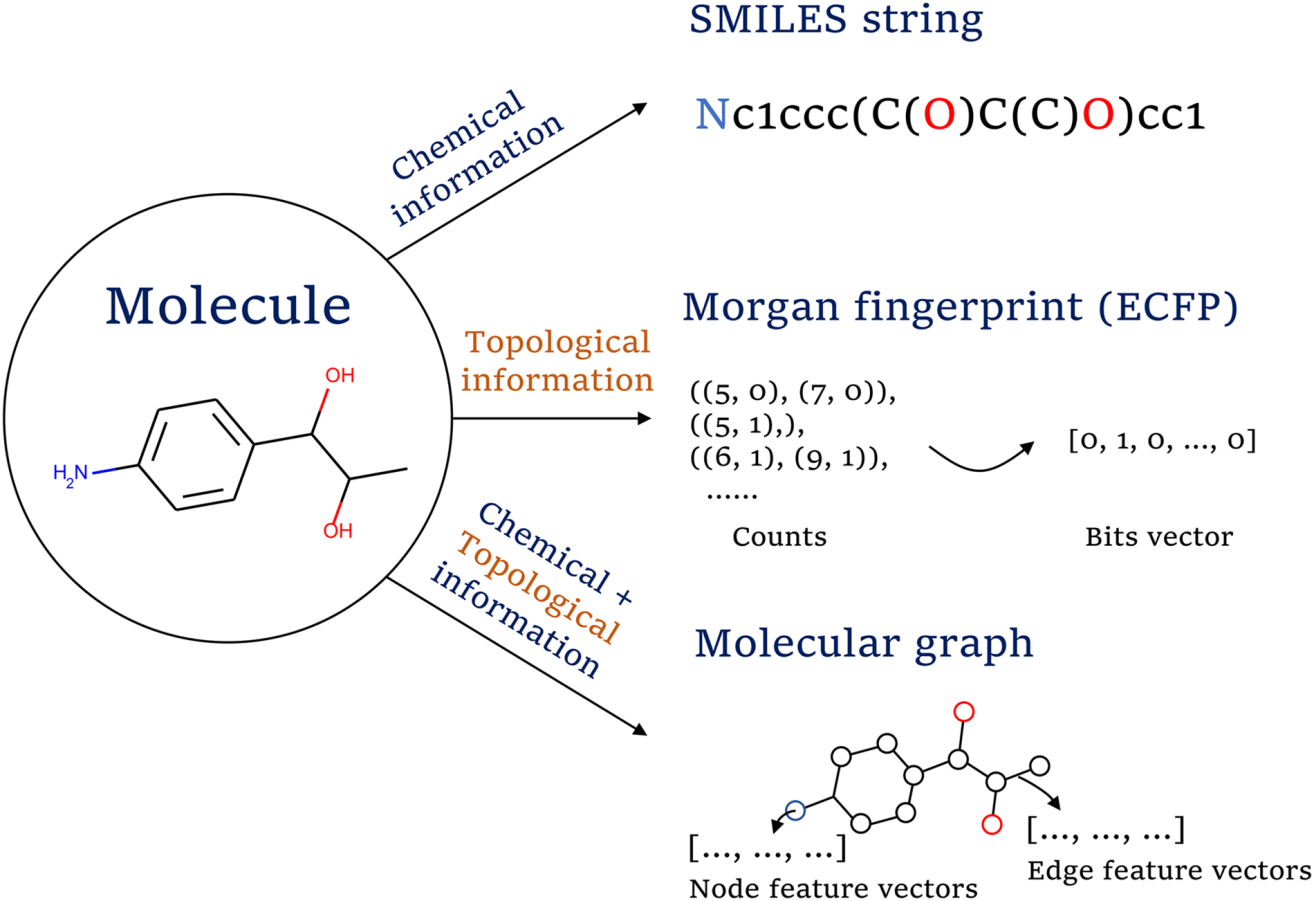
Molecular representations. SMILES string, Morgan fingerprint (Extended-connectivity fingerprint, ECFP), and molecular graph representations of a sample molecule

Molecular graphs (Coley et al., 2017; De Cao and Kipf, 2018; You et al., 2018) encode the topological structure of molecules naturally and intuitively by regarding atoms as nodes, bonds as edges, and atom types and bond types as node and edge features, respectively. However, a graph with n nodes can be represented by n! equivalent adjacency matrices, which makes it expensive for modeling a graph reconstruction problem. Thus, graph matching algorithms (Simonovsky and Komodakis 2018) and topological sorting algorithms (You et al. 2018) are first required due to the computational complexity. An example of the above three types of molecular representations is shown in Fig. 1.

The other challenge is the design of the objective function to be optimized. The QED, log*P*, and SAscore are widely used for measuring molecular properties. However, these knowledge-based or experience-based metrics do not work well for all drug molecules. Thus, optimizing inappropriate properties may lead to many false-positive results, i.e. molecules with high optimized property scores may have low drug potentials. On the contrary, some molecules with high drug potentials that do not meet these optimized property rules may be ignored. Thus, an effective method for molecular quality assessment (QA) on drug potentials is imperative in the RL-based molecular generation framework.

In this study, we propose an iterative refinement framework QADD (quality assessment-based drug design), which combines a multiobjective deep RL molecular generator with a novel molecular QA discriminator on drug potentials, to optimize multiple molecular properties iteratively. Inspired by protein QA (Sanyal et al. 2020), we embed the molecular QA module into the molecular generation model and train a graph neural network (GNN) model to score the quality on drug potentials of molecules, where the quality score is used as one of the reward functions of the RL model. To better preserve the rich structural information of molecules, we choose molecular graphs as the molecular representations. Considering that traditional neural networks cannot handle non-Euclidean data like graphs, we exploit GNNs (Bruna et al. 2014; Kipf and Welling 2017) to train the molecular QA model, where GNNs can effectively encode the structural information of molecules and automatically learn abstract representations using graph convolutions.

Furthermore, since the RL-based training pipeline is iteratively converging, updating the QA module to more effectively recognize the RL model’s generation preference during the iterative process is necessary. Thus, we retrain the molecular QA model several times when receiving a sufficient number of molecules from the RL model as negative samples for the QA model. Due to the expansion of these specific negative samples, this process will further improve the discriminative ability of the QA model and help improve the quality of the generated molecules in the next iteration.

To deal with the multiobjective optimization problem, we adopt a multiobjective deep RL method to optimize multiple molecular properties including QED, SAscore, and quality scores predicted by our molecular QA module in parallel. Considering the chemical space is discontinuous, we apply the DQN, a value-based RL method, to estimate the action-value function under different action selection strategies. Since DQN does not require a fixed-dimensional action space, it is particularly suitable for discontinuous space search. Then, we choose appropriate strategies according to the action-value function learnt from DQN model to optimize the molecular properties.

In general, drugs bind to a biological target to function as an inhibitor against a disease. Thus, we apply QADD to generate novel molecules with high binding affinity to the biological target, i.e. DRD2 (dopamine receptor type 2). DRD2 is an essential antipsychotic therapeutic target to treat schizophrenia, Parkinson’s disease, and psychotic disorders (Beaulieu and Gainetdinov, 2011; Howes et al., 2012; Wang et al., 2018), and proved to exert oncostatic effects on several cancers (Bakadlag et al., 2019; Tung et al., 2022). In this study, the DRD2 binding affinity of a molecule is estimated by a binary classification model which is trained to classify DRD2 binding molecules from non-binding molecules by fine-tuning the molecular QA model with binding affinity data of DRD2. The predicted binding affinity score serves as a reward function of the proposed multiobjective strategy, which is optimized to generate DRD2-specific molecules.

## 2 Materials and methods

### 2.1 Benchmark datasets

#### 2.1.1 QA dataset

In this study, we construct a benchmark dataset with 262 859 molecules consisting of 154 000 positives and 108 859 negatives. The positives are considered to have high drug potentials, while the negatives have lower ones. The 154 000 positive samples are randomly sampled from the ChEMBL database and they are bioactive molecules with high drug potentials. While the 108 859 negative samples are extracted from the previous RL experiments guided by QED and SAscore rewards after functional groups modification (Supplementary Fig. S1). These negative samples are filtered by QED < 0.605 and SAscore > 2.797 to ensure that they are nondrug-like molecules. We randomly split these molecules into the training and validation set with a ratio of 7:3. The distributions of different properties of these molecules in the benchmark dataset are shown in Supplementary Fig. S4.

Moreover, we construct an independent test set with 20 000 molecules consisting of 10 000 positive samples and 10 000 negative samples. These samples are extracted in the same way as the samples in the training set, and there is no overlap between the training dataset and the independent test set. The benchmark dataset is used to train the molecule QA model and the independent test set is used to evaluate the trained QA model.

#### 2.1.2 DRD2 binding affinity dataset

The DRD2 binding affinity dataset is extracted from ExCAPE-DB (Sun et al. 2017), and the molecules in the dataset are split into 8323 positive molecules (pIC50 ≥ 5) and 343 203 negative molecules (pIC50 < 5). We construct a benchmark dataset with all 8323 positives and randomly sampled 42 000 negatives. We randomly split these molecules into the training, validation, and test set with a ratio of 7:2:1 for DRD2 binding affinity prediction models.

### 2.2 GNN-based molecular QA

In our QADD pipeline, we apply a Graph Isomorphism Network (GIN; Xu et al., 2018) framework for molecular QA. GIN is one of the most powerful neighborhood aggregation-based GNNs. It takes the adjacency matrix, node feature matrix, and labels of a graph as the input, and outputs the embedded features of the graph throughout a readout layer. This model is characterized by the effective representation of the graphs and great discriminative power, and thus is suitable for modeling the molecular classification task.

The node embeddings are updated according to the information of the node and its neighboring nodes as follows: where $h_{i}^{\left(l\right)}$ represents the embeddings of node i in the lth layer, N(i) represents the set of neighboring nodes of the node i, ε is a hyperparameter which determines the weight of $h_{i}^{\left(l\right)}$, and MLP represents a nonlinear transformation.


(1) $$h_{i}^{\left(l+1\right)}=\mathrm{MLP}\;\left(\left(1+\varepsilon \right)h_{i}^{\left(l\right)}+\sum_{j\in N(i)}h_{j}^{\left(l\right)}\;\right)$$

The embeddings of the graph can be readout from the embeddings of all the nodes on the graph as follows: where G represents the graph, and n represents the number of the layers.


(2) $$h_{G}=\mathrm{CONCAT}\;\left(\sum_{i\in G}h_{i}^{\left(l\right)}\;|\;l=0,1,\ldots ,n\right)$$

The readout layer sums the node embeddings of each layer, and then concatenates the information of these layers to obtain the final graph embeddings.

We introduce a rank-based QAscore to normalize the scores predicted by the molecule QA model. All the molecules in the benchmark dataset are scored by the trained QA model and pooled to build a score library. The QAscore of a new molecule is determined by the ranking of its predicted score in the library as follows: where x represents the ranking of the molecule, n and m represent the number of positive samples and negative samples in the library, respectively. The numerical range of the QAscore is [0,1]; the higher the score is, the better the molecular quality is.


(3) QAscore= 0.5+n-x2n       x≥n 0.5+n-x2m       x<n

### 2.3 Deep RL

In the QADD pipeline, we model the molecular generation under the framework of Markov decision process (MDP), which is optimized using DQN algorithm.

General computational molecular generation can be mathematically modeled as a MDP: M(S, A, Pa(s,s′),Ra(s,s′)), where S represents the state space; A represents the action space; Pa(s,s′) and $Ra\left(s,s^{\prime }\right)\;$represent the probability of the transition and reward from the state s to the state $s^{\prime }\;$under the action a, respectively.

**Figure 2:**
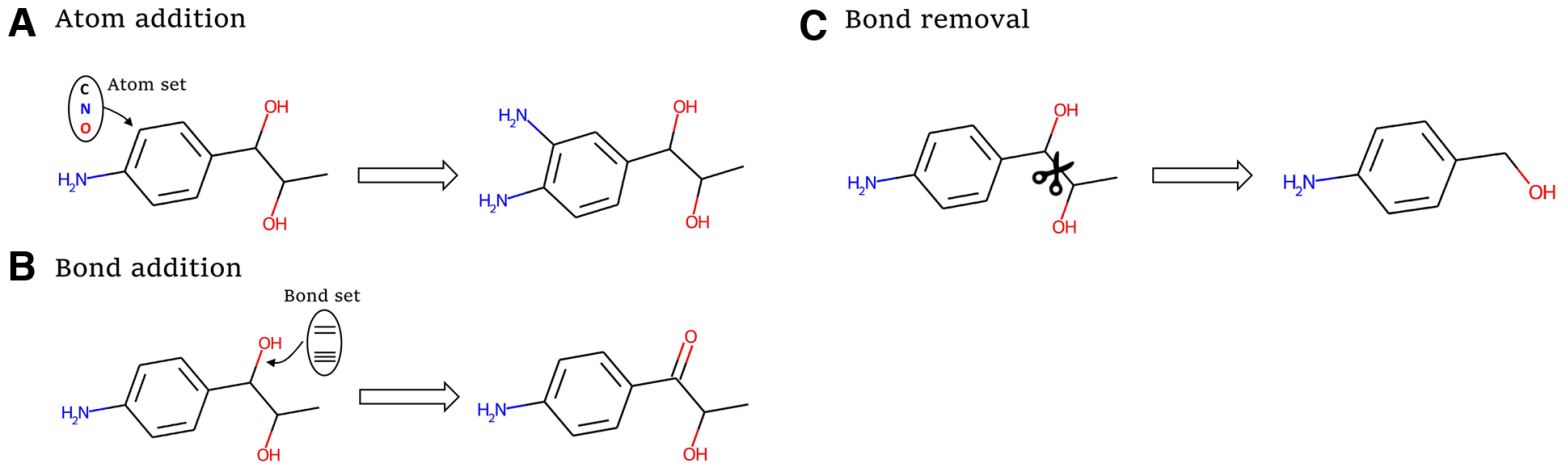
Illustration of the three main types of one-step valid modifications in the action space. (A) Atom addition: randomly selects an atom from the atom set and adds a covalent bond between the atom and a random atom with implicit hydrogens on the target molecule (note that the bond type is limited by the implicit hydrogens of the connected atoms); (B) Bond addition: adds a valid covalent bond between two random atoms with implicit hydrogens on the target molecule; (C) Bond removal: removes a random covalent bond (except bonds on the aromatic ring) on the target molecule, and the potential free atom or small fragment will be removed as well

The action space A includes all the calculated one-step valid modifications based on the current state s, and is divided into four types: atom addition, bond addition, bond removal, and remaining unchanged, as shown in Fig. 2. The details of the MDP configures are given in the support information.

In the drug design task, the action spaces of molecules are discontinuous and not suitable for many classical RL algorithms like PPO (Schulman et al., 2017). To resolve this issue, DQN is used and it pays attention to the action that maximizes the Q function, regardless of whether the dimension of the action space is fixed or not. DQN is a deep value-based RL model and it has been proved effective in molecular properties optimization (Mnih et al., 2015; Zhou et al., 2019). To optimize multiple molecular properties, we adopt a multiobjective framework motivated by (Liu et al., 2014) that allows building an individual Q network for each objective function to prevent information loss in the MDP process. These Q networks built for different objective functions have the same structure and are trained in parallel, while the predicted Q functions are the weighted sum for action selection.

In DQN, the action-value function (called as Q function) is defined as the conditional expectation of the discount return: where π represents the action choosing strategy, $U_{t}$ represents the discount return (the detailed definition is given in the support information),${\;s}_{t}$ and${\;a}_{t}$ represent the state and action at the time *t*, respectively.


(4) $$Q_{\pi }\left(s_{t},\;a_{t}\right)=\;E\left(\left.U_{t}\;\right|{\;s}_{t},a_{t}\right)$$

The action choosing strategy π is to maximize the Q function:


(5) $$Q\left(s_{t},\;a_{t}\right)=\underset{\pi }{\max\;}Q_{\pi }\left(s_{t},\;a_{t}\right)$$

The Q-function is estimated using the temporal difference (TD) algorithm (Tesauro, 1995), where the TD error is calculated through Optimal Bellman Equations and Monte Carlo Simulation as follows: where ω represents the parameters of the current Q network, and it is updated through backpropagation. γ represents the discount factor, the closer it is to 0, the more the model focuses on short-term returns.


(6) $$\mathrm{TDerror}=Q\left(s_{t},\;a_{t};\omega \right)-(R_{t}+\underset{\;a\;\in \;A}{\gamma \cdot \max\;}Q\left(s_{t+1},\;a;\omega \right))$$

An ε-greedy strategy is introduced to provide the uncertainty during exploration, it allows stochastic action selection to improve the diversity and jump out of the local optima. The action $a_{t}$ is chosen randomly with the probability of ε, and is chosen to maximize the Q function in the action space A with the probability of 1-ε. DQN consists of two Q networks, an eval Q network and a target Q network with the same structure. The parameters of the target Q network are updated slower to improve the network stability. An experience replay policy (Fang et al., 2017) is applied to break the correlation among the training data, where the training data is randomly selected from the memory that stores previous states during the model training. The details of DQN are given in the support information.

### 2.4 Iterative refinement framework

**Figure 3:**
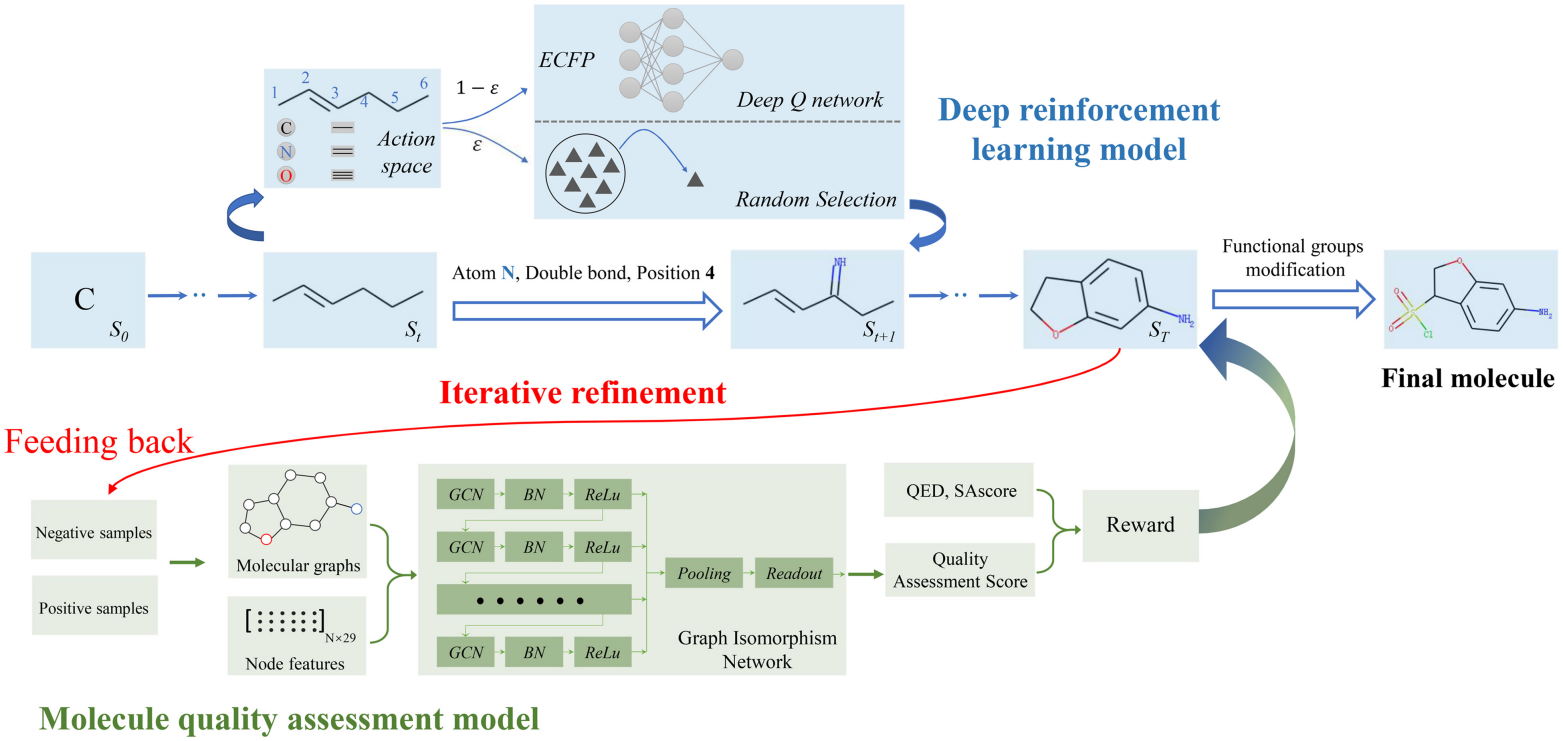
The pipeline of the proposed method QADD. The multiobjective deep RL model estimates the value function of the generated molecules and chooses the most appropriate action at each step to maximize the discounted return. The QAscore scored by the molecule QA model serves as one of the reward functions of the multiobjective deep RL model, whose generated molecules are fed back to retrain the GNN-based QA model iteratively. Finally, the generated molecules are further modified using the functional group modification

The QAscore is applied as one reward function of the deep RL model to guide the generation of high-quality molecules. However, the negative samples in the benchmark dataset may not cover all molecules with the low drug potentials due to the huge chemical space. Considering that the model will have its preference, we thus propose an iterative refinement framework that allows molecules generated by the RL model to be fed back to retrain the QA model, as shown in Fig. 3. These generated molecules with a low quality (QED < 0.605 and SAscore > 2.797) are updated as negative samples after adding functional groups, while the positive samples remain the same. Then, the QAscores of generated molecules are updated by the retrained QA model. The above process is repeated until the success rate of generated molecules no longer improves or the retrained QA model does not converge. Finally, the generated molecules are further modified using the functional group modification (Supplementary Fig. S2).

### 2.5 Experimental settings

#### 2.5.1 Objective functions

We apply QED, SAscore and our proposed QAscore into the reward function of the deep RL model. Table 1 lists the reward functions used in the different methods. Generative models including BIMODAL (Grisoni et al., 2020) and REINVENT (Olivecrona et al., 2017) learn the distribution of the molecules and do not apply specific reward functions.

- QED: Quantitative estimation of drug-likeness, which is the normalized weight of eight molecular descriptors including molecular weight (MW), log*P*, the number of hydrogen bond donors and acceptors, molecular polar surface area, number of rotatable bonds, number of aromatic rings and number of structural alerts with a range of [0,1]. A high QED suggests a high drug-likeness.
- SAscore: Synthetic accessibility score, which takes the frequency of occurrence of compound substructures in the PubChem database and structural complexity into account, it is scaled into [1,10]. The closer to 1 the SAscore is, the more synthesizable the molecule is.
- QAscore: Molecular QA score, which is a normalized rank-based score predicted by our QA model with a range of [0,1]. A high QAscore suggests a high molecular quality for drug potentials.

To demonstrate the advantage of QADD, we compare it with four recently published baseline methods:

- **molDQN** (Zhou et al., 2019): it introduces a DQN framework with double Q-learning and randomized value functions. molDQN encodes molecules as Morgan fingerprints and jointly optimizes the QED and log*P* properties of molecules.
- **MARS** (Xie et al., 2021): it is a state-of-the-art approach for multiple molecule properties optimization. MARS combines Markov chain Monte Carlo sampling and GNN to optimize the bio-activity, drug-likeness, and synthesizability of molecules.
- **BIMODAL** (Grisoni et al., 2020): it proposes a bidirectional strategy for molecular SMILES strings generation. BIMODAL combines a forward and a backward RNN to predict a joint probability distribution of bioactive molecules from the ChEMBL dataset.
- **REINVENT** (Olivecrona et al., 2017): it is an RL-guided molecular SMILES strings generation approach based on the RNN model. REINVENT applies a policy-based RL model to fine-tune the pretrained RNN generative model to generate molecules with desirable properties.

In addition, we compare QADD with other three variants of QADD using different reward functions:

- QADD-1st QA: it jointly optimizes QED, and QAscore of the QA model in the 1st iteration.
- QADD-2nd QA: it jointly optimizes QED, and QAscore of the QA model in the 2nd iteration.
- QADD-3rd QA: it jointly optimizes QED, and QAscore of the QA model in the 3rd iteration.
- QADD: it jointly optimizes QED, SAscore, and QAscore of the QA model in the 3rd iteration.

#### 2.5.2 Evaluation metrics

We evaluate the generated molecules on the following five metrics:

- **Validity:** it is the percentage of molecules that satisfy the chemical validity.
- **Success rate:** it is the percentage of molecules with scores higher than the average QED and SAscore (QED>0.605 and SAscore<2.797) of molecules in the ChEMBL database.
- **Diversity:** it is used to assess the similarity within generated molecules and calculated as $1-\sum_{x,y\in S;\;x\neq y}\mathrm{sim}\left(x,y\right)/n\left(n-1\right),\;$where S represents the set of generated molecules, n represents the number of molecules in S, and sim represents the Tanimoto similarity of the Morgan fingerprints of the two molecules x and y.
- **Novelty:** it is used to assess the similarity between generated molecules and molecules deposited in the ChEMBL database. It is calculated as $1-\mathrm{mean}\left(\mathrm{sim}\left(x,y\right)\;\right|\;x\in S,\;y\in S^{\prime })$, where $S^{\prime }$represents the molecules in the database.
- **QAscore and QED**: they are used to evaluate the effectiveness of the optimization method and the quality of the generated molecules, respectively.

For the molecular QA model, we also design a CNN-based model to demonstrate the effectiveness of GNNs. The CNN-based model takes the SMILES strings as the input, which are encoded to numeric vectors and then passed through an embedding layer, two convolutional layers, a batch normalization layer, three fully connected layers, and a Softmax layer to output the predicted scores. We evaluate the performance of molecular QA model and DRD2 binding affinity prediction model using the area under receiver operating characteristic curve (auROC) and area under precision-recall curve (auPRC).

## 3 Results

We demonstrate that our proposed QADD generates the molecules with high molecular quality. The proposed molecular QA model in QADD achieves a high performance for assessing the drug potentials of generated molecules. The ablation studies on the combination of different properties demonstrate the effectiveness of the designed multiobjective optimization strategy in QADD. And QADD achieves superior performance to other baseline methods for generating molecules with high drug potentials. Moreover, QADD successfully generates novel molecules with high binding affinity to DRD2 target.

### 3.1 Performance of the molecular QA model

We first evaluate the trained QA model on the independent test set. As shown in Supplementary Fig. S5A and B, both the loss and accuracy of the proposed GNN-based QA model on the training set and validation set converge fast. We further evaluate the trained QA model on the independent test set, the QA model yields an accuracy, sensitivity, specificity, and MCC (Matthews correlation coefficient) of 0.9943, 0.9978, 0.9908, and 0.9886, respectively. We can see that the proposed QA model yields a high performance.

**Table 1.** The applied objective functions of different methods.^a^

Method	QED	SAscore	QAscore	Others
molDQN	**√**	**√**		
MARS	**√**	**√**		**√**
QADD-1st QA	**√**		**√**	
QADD-2nd QA	**√**		**√**	
QADD-3rd QA	**√**		**√**	
QADD	**√**	**√**	**√**	

a 1st QA, 2nd QA, and 3rd QA represent the 1st, 2nd, and 3rd iteration of our iterative refinement model respectively, and QADD represents the third iteration model with an extra SAscore reward function.

**Figure 4:**
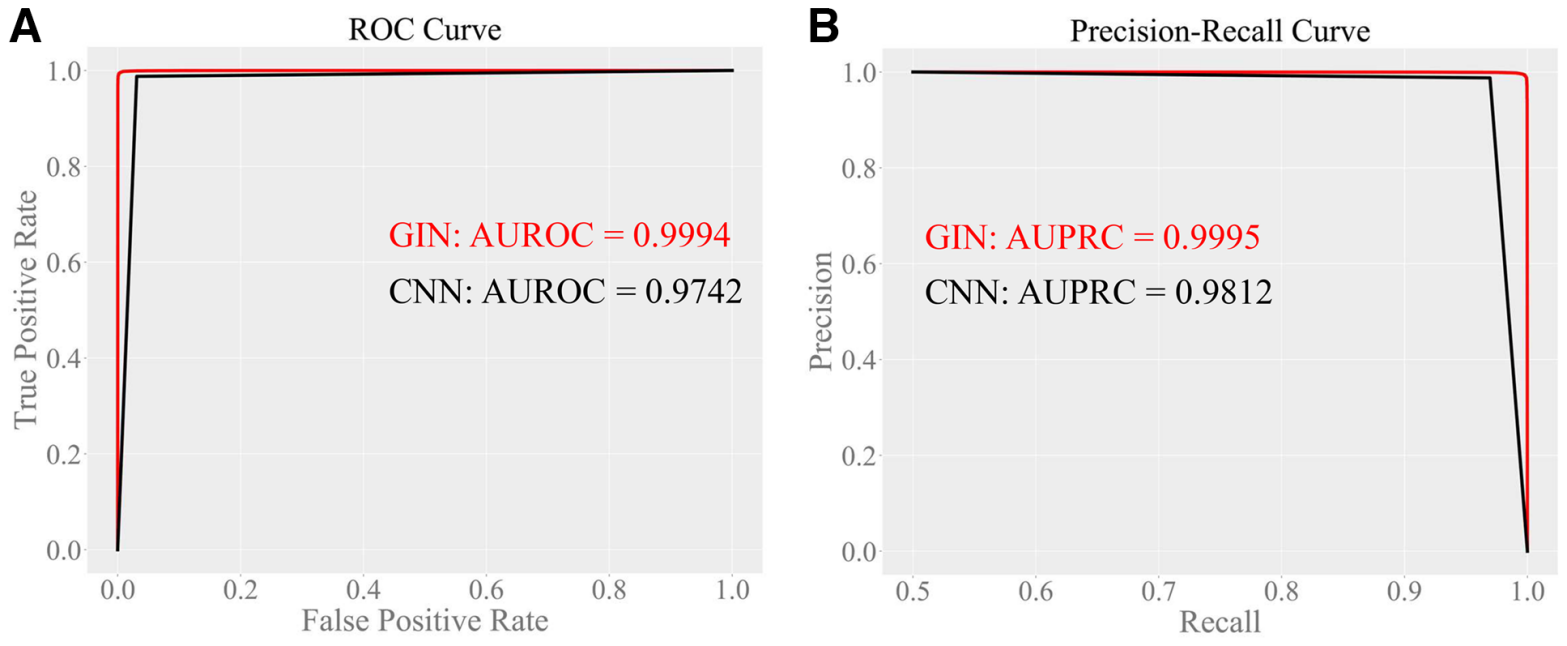
The performance of molecular AQ model. The ROC curve (A) and precision-recall curve (B) of the third iteration QA model with CNN and GIN model on the independent test set

To demonstrate the power of GINs, we compare the GIN-based QA model with a CNN-based QA model on the same training and test set. The ROC curve and PR curve are shown in Fig. 4A and B. We can see that the performance of the GIN-based QA model is superior to the CNN-based QA model. The GIN-based QA model achieves an auROC of 0.9994 and auPRC of 0.9995, which is higher than the auROC 0.9742 and auPRC 0.9812 of the CNN-based QA model, respectively. The results demonstrate the effectiveness of GNNs for the molecular QA task.

### 3.2 Performance of QADD with different combinations of the reward functions

In this study, we present a multiobjective RL to jointly generate molecules with multiple desired properties. We evaluated different combinations of reward functions derived from multiple properties in our RL model. In addition to the reward functions introduced in Section 2.4, we introduce other two reward functions derived from the MW and Phenyl-containing group, and one weighted sum of multiple rewards:

- MW reward function: a normalized reward that reflects the distance between the target MW and a predefined threshold (set as 500 in the experiments) without any knowledge.
- Phenyl-containing (P) group reward function: a discrete reward that can only be 0 or 1. The value of 1 means the target molecule contains at least 1 phenyl group and 0 means the opposite.
- A weighted sum of multiple reward functions: it combines the reward functions derived from QED, SAscore, and QAscore with the same weight.

Here, the generated molecules guided by the MW reward function are considered to be a blank control, i.e. randomly generated molecules without the guidance of any property knowledge. The phenyl-containing group reward function is introduced to investigate whether molecules containing the target substructure can be generated. The weighted sum of multiple rewards is compared to demonstrate the effectiveness of the multiobjective optimization strategy used in QADD model.

**Figure 5:**
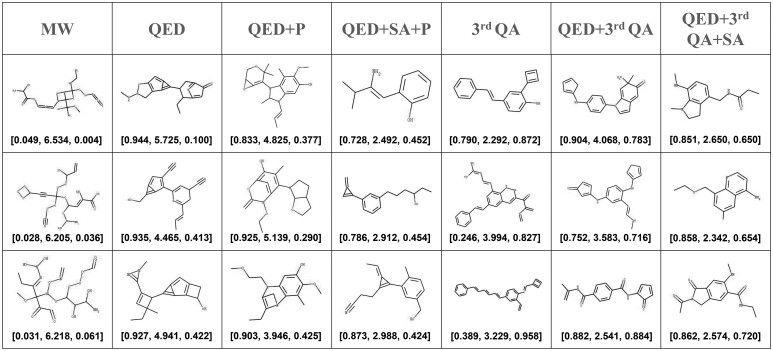
Sampled molecules generated by QADD with different combinations of reward functions. “MW” represents the molecular weight reward function; “P” represents the phenyl-containing reward function; “SA” represents the SAscore reward function; “third QA” represents the QA reward function in the third iteration. The evaluation metrics of the molecules are shown in the bottom in the format of [QED, SAscore, QAscore]

As shown in Fig. 5, the generated molecules with different reward functions are illustrated. We can see that molecules generated with a single MW reward are obviously nondrug like, revealing the low quality of generated molecules without the guidance of the knowledge-based metrics. Molecules generated with a single QED reward are observed to have simple structures. The potential reason is that the preference of the two metrics makes molecules with simple structures obtain higher QED and SA scores. Molecules generated using a single QA reward have longer carbon chains than other single rewards, but they also appear to be nondrug like due to the limited number of training samples compared to the whole huge chemical space. Thus, QADD combines the QED and QA rewards as the multiobjective function. Although the QA reward does not work well alone, we find the QED and QA rewards are highly complementary. The generated molecules with a combined reward of QED and QA appear to be drug like. The property distributions of the generated molecules under different combinations of reward functions are shown in Supplementary Fig. S6.

Furthermore, we evaluate the molecules generated by two different multiobjective optimization strategies. One converts the multiobjective optimization into a single-objective optimization with the weighted sum of the multiple reward functions. The other adopts the multiobjective framework which builds a Q network for each objective reward function. Both approaches optimize the same molecular properties consisting of QED, SAscore, and QAscore. The performance on quantitative evaluation metrics is given in Table 2, we can see that the multiobjective framework in QADD generates molecules with a higher novelty and diversity, and close success rate to the approach using the weighted sum of multiple reward functions.

### 3.3 QADD outperforms baseline methods for molecules generation

We compared our method QADD with four baseline methods molDQN, MARS, BIMODAL, and REINVENT on 1000 generated molecules using the widely used metrics validity, success rate, novelty, diversity, QAscore, and QED. The results of the four baseline methods are obtained by running their source codes, and we extract the result of the last 1000 generated molecules in all 5000 episodes to keep fairness.

We first compare QADD with four baseline methods molDQN, MARS, BIMODAL, and REINVENT on the evaluation metrics. As shown in Table 3, we can see that QADD and molDQN yield a 100% validity, which is higher than 99.7% of MARS and BIMODAL, and REINVENT yields the lowest validity of 94%. Of other three metrics, QADD yields the highest success rate of 0.8489 ± 0.0192, which is higher than 0.017 of molDQN and 0.798 of MARS. For other two metrics, QADD yields a novelty of 0.3410 ± 0.024 and diversity of 0.6139 ± 0.0291, which are both close to the highest novelty 0.360 of molDQN, but lower than the highest diversity of 0.7552 of REINVENT due to the sequence-based generative model. The results demonstrate that QADD can yield stable performance across the four metrics.

**Table 2.** Performance comparison of applying the weighted sum of multiple rewards and the multiobjective framework used in QADD on the quantitative evaluation metrics.^a^

Method	Validity (%)	Success rate	Novelty	Diversity
Weighted sum^b^	**100**	0.8476 ± 0.0328	0.2817 ± 0.0653	0.5865 ± 0.0292
Multiobjective	**100**	**0.8489 **±** **0.0192	**0.3410 **±** **0.0240	**0.6139 **±** **0.0291

a Three independent experiments were run for each method to calculate the mean and standard deviation.

b Converting multiple objectives into a single objective function by weighted combination with the same weights for different objective reward functions.

**Table 3.** Performance comparison of different methods on the quantitative evaluation metrics.^a^

Method	Validity (%)	Success rate	Novelty	Diversity
molDQN	**100**	0.0170	0.3609	0.5310
MARS	99.7	0.7980	0.3332	0.6413
BIMODAL	99.7	0.2858	0.3144	0.7201
REINVENT	94.0	0.3750	0.3076	**0.7552**
QADD-1st QA	**100**	0.2693 ± 0.1218	0.3570 ± 0.0227	0.5771 ± 0.0765
QADD-2nd QA	**100**	0.0760 ± 0.0521	0.3928 ± 0.051	0.7064 ± 0.0651
QADD-3rd QA	**100**	0.5166 ± 0.0827	**0.4489 **±** **0.013	0.6645 ± 0.0347
QADD	**100**	**0.8489 **±** **0.0192	0.3410 ± 0.024	0.6139 ± 0.0291

a The results of molDQN, MARS, BIMODAL, and REINVENT are obtained by running their source code. The results of the 2nd QA, 3rd QA, and QADD are the mean and standard deviation calculated by running three times. Bold face indicates the method yields the best result across the compared methods.

We further investigate the impact of the QA model from different iterations on the generated molecules. As can be seen from Table 3, the success rate and novelty of the generated molecules by QADD with the third-iteration QA are significantly improved compared with the previous two iterations, demonstrating the effectiveness and correctness of the iterative refinement model. Moreover, the success rate exceeds 0.5 without the SAscore objective function, demonstrating the QA model covers some molecular properties to a certain extent. Adding SAscore reward function can yield a high success rate of 0.8489. However, we also observed that adding the SAscore reward function is not always a good choice in our experiments, because it tends to oversimplify the molecular structure, resulting in a lower diversity and novelty. These results show that we need make a tradeoff among different performance metrics when using different QA model and reward functions.

We further compare QADD with other methods on the metrics derived from different objective functions. As shown in Table 4, we can see that QADD successfully optimized QED and QA with promising performance on QED mean, QA max, and QA mean. Interestingly, we observe molecules generated by MARS have high QA scores. One potential reason is that MARS generates molecules by assembling frequently appearing fragments, which are basically consistent with the substructures of positive samples in our benchmark dataset. However, fragment-based methods like MARS also have certain limitations such as the low novelty due to the inability of atom-resolution editing. Moreover, BIMODAL and REINVENT yield high diversity due to the exploration ability of the sequence-based generative model, but they both suffer to the limited novelty.

### 3.4 QAscore serves as a discriminative label for generated molecules

**Figure 6:**
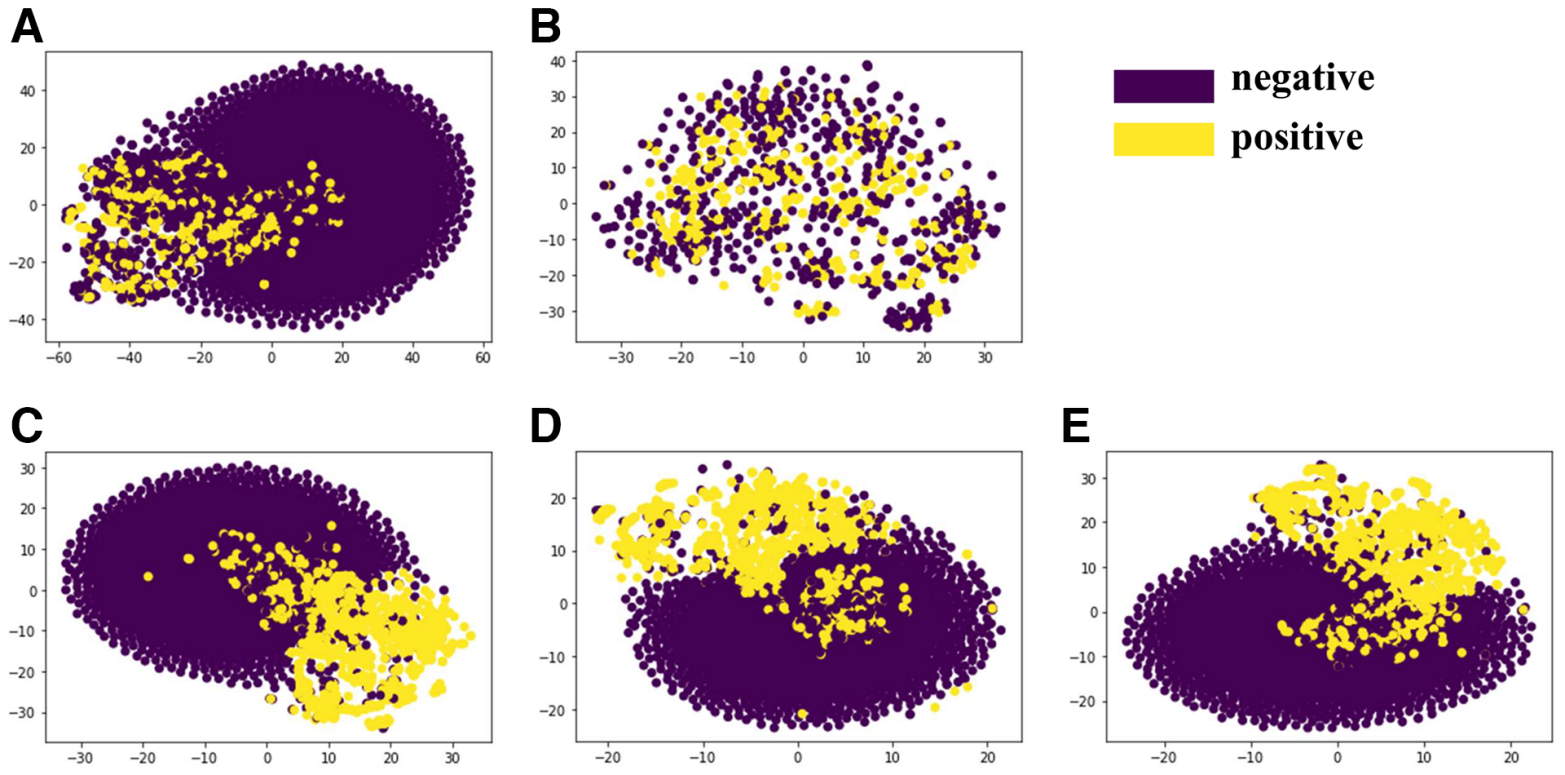
t-SNE visualization of generated molecules with labels defined by different criterion. (A) 5000 molecules annotated by SR label; (B) the last 1000 molecules annotated by SR label; (C) 5000 molecules annotated by the 1st iteration QA label; (D) 5000 molecules annotated by the second iteration QA label; (E) 5000 molecules annotated by the third iteration QA label

To demonstrate the quality of generated molecules, we visualize the distributions of the generated positive and negative molecules. As shown in Fig. 6, the 5000 molecules in the final iteration (QADD) are visualized by t-distributed stochastic neighbor embedding (t-SNE) (van der Maaten and Hinton, 2008), where the labels are defined according to three different criteria: (i) SR label, positive molecules have QED>0.605 and SAscore<2.797; (ii) QA label, positive molecules have QAscore>0.5, and the ECFP is applied as the molecular descriptor. In Fig. 6A and B, the positive and negative molecules are annotated by the SR label, we can see that the positive and negative samples are relatively separated in Fig. 6A while they are mixed together in Fig. 6B. The reason may be that the molecules generated in the last 1000 episodes are of high quality or close to high quality, resulting in the difficulty to distinguish by the SR label.

**Table 4.** Performance comparison of different methods on the objective function metrics.^a^

Method	QAscore max	QAscore mean	QED max	QED mean
molDQN	0.4890	0.3671	0.9433	0.7610
MARS	0.9779	0.7758	0.9401	0.7464
BIMODAL	**0.9990**	**0.8008**	**0.9455**	0.5414
REINVENT	0.8674	0.4619	0.9217	0.5250
QADD-1st QA	0.6475 ± 0.1176	0.4239 ± 0.0156	0.9331 ± 0.0028	0.7167 ± 0.0269
QADD-2nd QA	0.7288 ± 0.1474	0.4735 ± 0.0996	0.9281 ± 0.0172	0.7204 ± 0.1063
QADD-3rd QA	0.9797 ± 0.0037	0.7793 ± 0.0311	0.9399 ± 0.0070	0.7830 ± 0.0116
QADD	0.9415 ± 0.0291	0.7265 ± 0.0230	0.9239 ± 0.0012	**0.7852 **±** **0.0080

a The “max” and “mean” represent the maximum value and mean value of the molecules, respectively. Bold face indicates the method yields the best result across the compared methods.

In Fig. 6C–E, positive and negative molecules are annotated by QA label, we can see that the positive molecules are clearly separated from the negative molecules, indicating that the metrics QED, SAscore, and QAscore have certain preferences to high-quality molecules. In addition, the generated molecules of other methods and QADD are visualized using t-SNE in Supplementary Fig. S8, we can observe that molecules generated by different methods are separated into different regions, implying that different drug design methods have different preference on generated molecules and there is a strong complementarity among these methods.

### 3.5 QADD is able to design candidate molecules binding to specific biological targets

We further demonstrate that QADD can generate novel molecules with high binding affinity to biological targets, here DRD2 is chosen as the biological target. We fine-tune the 1st QA model to classify binding molecules and nonbinding molecules of DRD2 using the constructed DRD2 binding affinity dataset, where the fine-tuned model is denoted as QADD-affinity. As shown in Table 5, we can see that the DRD2 binding affinity prediction model yields high performance, which is a little better than REINVENT.

**Table 5.** Performance of the DRD2 binding affinity prediction model on the test set of the DRD2 binding affinity dataset.

Set	REINVENT	QADD-affinity
Accuracy	0.98	0.99
Sensitivity	^a^	0.98
Specificity	^a^	0.99
Precision	0.97	0.99
Recall	0.82	0.88
MCC	^a^	0.96
AUROC	1.00	1.00
AUPRC	^a^	0.99

a Means this metric is not reported in the original paper.

**Figure 7:**
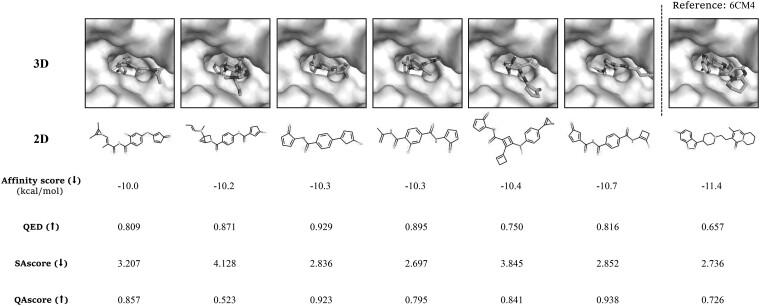
The docking structure of the DRD2 protein and six generated molecules (blue) by QADD with top predicted binding affinity, where the reference complex is 6CM4 complex between the ligand 8NU and DRD2. A lower affinity score indicates a higher binding affinity

In this experiment, the predicted scores of the DRD2 binding affinity prediction model serve as an additional reward function of our QADD framework, and the other settings remain the same as the previous experiments. We evaluate the binding affinity of the generated molecules binding to DRD2 in the last 100 episodes by the molecular docking tool (AutoDock Vina; Eberhardt et al., 2021) and compare them against the experimentally resolved complex structure 6CM4 (Wang et al., 2018) between DRD2 and the ligand 8NU, which is denoted as the reference ligand (Rose et al., 2016). We illustrate the docking structure of the DRD2 protein with six generated molecules according to the top binding affinity using Pymol (DeLano, 2002). As shown in Fig. 7. The average affinity score of the docking structures between the top 100 generated molecules and DRD2 reaches -9.4 kcal/mol. Of them, the top-10 affinity scores are below -10.0 kcal/mol (Supplementary Fig. S7). We can see that the generated molecules have good binding affinity, which is close to -11.4 kcal/mol of the resolved 6CM4 complex. The results demonstrate that our QADD model successfully generates molecules with high DRD2 binding affinity.

We further compare QADD with target-based multiobjective molecules generation method like MARS on the DRD2 binding affinity. Since MARS did not provide the DRD2 binding affinity prediction model, we applied the QADD-affinity as one of the objectives. The generated molecules in the last 100 episodes are used to calculate the evaluation metrics. As shown in Table 6, we can see that QADD yields higher success rate and lower average binding affinity score than MARS.

**Table 6.** Performance of the DRD2 binding affinity prediction model on the test set of the DRD2 binding affinity dataset.

Method	Success rate	Average binding affinity (kcal/mol)
QADD	**0.54**	**−9.4**
MARS	0.42	**−**8.6

We further evaluate the top-10 molecules (Supplementary Table S1) against the referenced ligand (8NU) in PDB structure 6CM4 on several metrics and ADMET (absorption, distribution, metabolism, excretion, and toxicity) properties. The ADMET properties are predicted by ADMETlab (Dong et al., 2018). As shown in Fig. 7, we observed that the top-10 molecules have better QED and QAscore, but lower SAscore than the reference ligand 8NU (Supplementary Table S2). In addition, the average score of the top-10 molecules is higher than the referenced ligand on all 31 ADMET properties (Supplementary Table S3), which indicates that these molecules may have lower in vivo toxicity and fewer damages to human health.

## 4 Discussion

In this study, we present a multiobjective deep RL-based method QADD with a novel QA module for *de novo* design of chemical molecules with desired properties. QADD shows promising performance in the *de novo* drug design task due to the followings:

1. The multiobjective RL method jointly optimizes multiple properties. Multiple objective functions are optimized in parallel, which avoids adding two or more potentially conflicting objective functions directly and the potential information loss.
2. The effective molecular QA model estimates good drug potentials for generated molecules. Previous methods perform optimization on molecular properties like QED, log*P*, and SAscore. Although optimization with the constraints of these metrics can distinguish some molecules with a low quality, the generated molecules still need be further improved. Our proposed QA model is an effective discriminative model that learns the distribution of molecules with high drug potential, which serves as an accurate guide for the RL generative model.
3. The essential iterative refinement framework improves the molecules iteratively. It gradually improves molecular properties by retraining the QA model on the generated molecules to cover more regions in the huge chemical space.
4. We apply QADD to generate novel molecules with high binding affinity to a biological target, where a binding affinity prediction model is designed to score target-specific binding affinity as one reward function of QADD.

However, as shown in Table 4, when integrating the QA model from different iterations, the generated molecules are preferred to be different on the quantitative metrics, no model can always yield the highest value across all the metrics. QADD variants that achieve high molecular quality appear to have low diversity and novelty. The exploration ability of the RL model maybe limited due to the multiple objective functions, thus the model is easily trapped in the local optimum, resulting in the low diversity and novelty. How to better balance and integrate the molecular quality and the similarity metrics is a potential future direction. Adding a structure similarity penalty into the RL generator is expected to be an effective way to alleviate the problem, but more powerful methods are needed to resolve the problem.

Furthermore, we use T-SNE to compare and visualize molecules generated by molDQN, MARS, and QADD (Supplementary Fig. S6). We observed that molecules are divided into three regions with little overlap, implying that different drug design methods have different preferences on generated molecules and there is a strong complementarity among these methods.

In this study, we only focus on the properties of 2D molecules, more information can be exploited like molecular 3D structure and molecular energy in future work. Novel objective functions derived from them should be used to design drugs to expect further improving the efficacy.

## 5 Conclusion

Computational design is a promising way to explore the whole huge chemical space to discover potential drugs. In this study, we propose an iterative approach QADD consisting of multiobjective deep RL and GNN-based QA module for *de novo* drug design. A multiobjective framework is designed to jointly optimize multiple properties instead of using a weighted sum of objective functions. We introduce a new metrics QAscore to assess the molecular quality on drug potentials by a GNN-based QA model, which is iteratively retrained on the molecules updated from DQN. In order to model the molecular generation preference of the network, QADD adopts an iterative refinement framework for molecule generation with high drug potentials scored by the QA model. Furthermore, we apply QADD to generate novel molecules with high binding affinity to a biological target. The results demonstrate the iterative multiobjective QADD approach has promising performance and is capable of generating molecules as promising drug candidates.

## Supplementary Material

btad157_Supplementary_Data
