# Supplementary material for: De novo drug design by iterative multiobjective deep reinforcement learning with graph-based molecular quality assessment
Source: Bioinformatics. 2023 Mar 24;39(4):btad157. doi: 10.1093/bioinformatics/btad157 (PMC10085518; doi:10.1093/bioinformatics/btad157)
Supplement: btad157_Supplementary_Data [file btad157_supplementary_data.pdf]

De Novo Drug Design by Iterative Multi-Objective Deep Reinforcement Learning with Graph-based Molecular Quality Assessment

Support information

1. Functional groups modification

Possible atoms in drugs are listed as follows: $\{H, C, N, O, F, P, S, Cl, Br, I\}$. However, the ways that atom *P* and *S* participate in forming a covalent bond are different from the simple rules for atoms like *C*, *N*, and *O*. Experimental results in the atomic-resolution reinforcement learning generative model show that adding *P*, *S*, and halogens leads to invalid molecules. Fortunately, these atoms occur in fixed patterns, which are called functional groups. We collected these functional groups to modify the molecules generated by our QADD model. The generated molecules are modified by a single substitution or a double substitution on *C*, *N*, or *O* atoms with enough implicit valence. The collected functional groups are shown in Figure S1.

For single functional group addition, the following steps are employed:

(1) Filter atoms (expect H atoms) with implicit valence >= 1;

(2) Add a 'Br' atom and a single bond linked with a random atom from (1);

(3) Replace 'Br' with common functional groups;

(4) Sanitize the final molecule.

Notice that all the functional groups showed in Figure S1 can form a single covalent bond with atoms with implicit valence >= 1 expect 'Thiocarbonyl' (The symbol * in Figure S1 hints the position to form the bond). So, we transfer the 'Thiocarbonyl' functional group from '=S' to '-C=S'. And the double functional groups modification is conducted by doing single functional group modification twice in sequence. The interaction between the modified two functional groups is ignored.

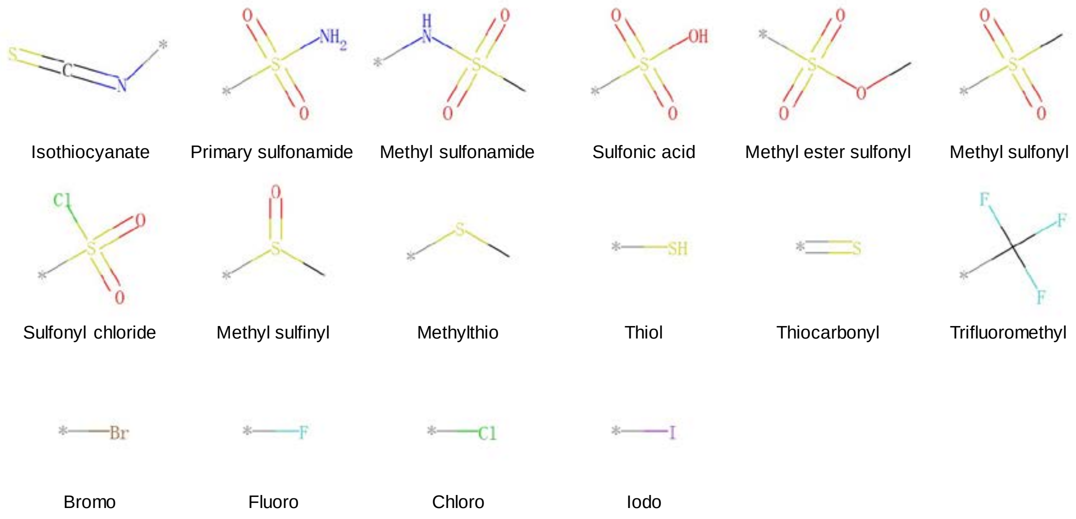

Figure S1. Common functional groups containing $\{F, P, S, Cl, Br, I\}$ atoms.

RL-based generated molecules may generate some irregular molecules, which can be corrected using prior knowledge on molecules. To demonstrate the added value of functional group modification, we illustrate the molecules generated by QADD before and after adding functional groups in Figure S2. Figure S2A displays the initial generated molecules using the atom set $[C, N, O]$, we can see that some irregular molecules do not conform to a real atomic type. To further improve the initially generate molecules, we randomly employ some functional group substitutions on the raw molecules. As shown in Figure S2, we can see the updated molecules are visually more

similar to real drug molecules than the raw molecules, demonstrating the necessity of adding the functional group substitutions on the raw generated molecules from RL models. The QED, SAscore, and QAscore distributions before and after functional groups modification are shown in Figure S3.

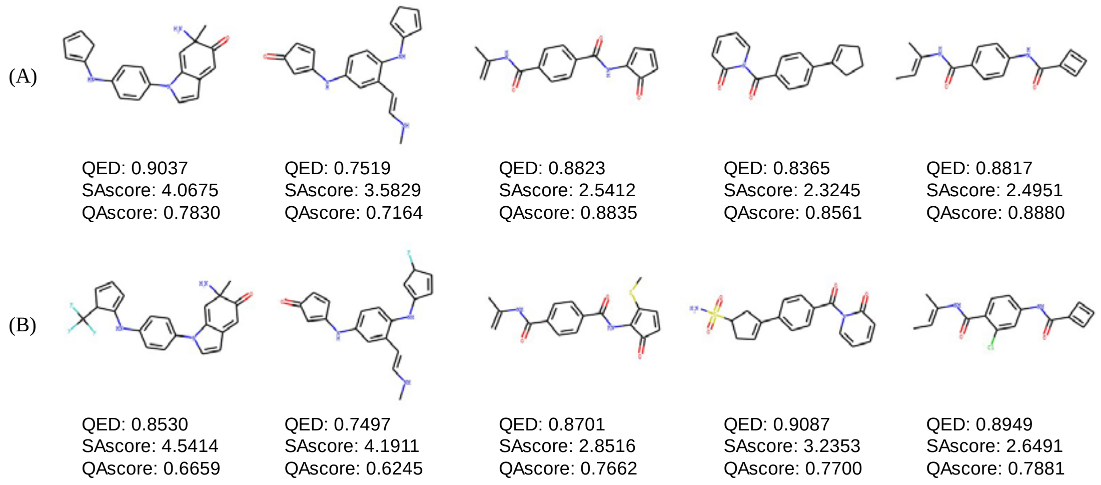

Figure S2. (A) Samples of initial molecules generated by QADD; (B) Samples of corresponding molecules generated after adding functional groups with other common atoms.

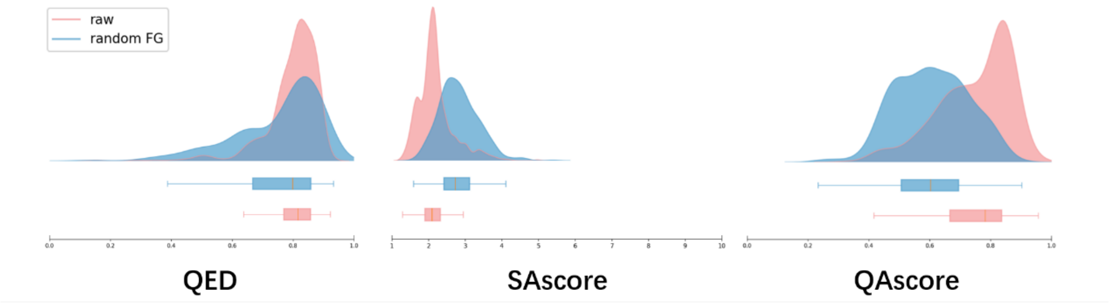

Figure S3. QED, SAscore, and QAscore distributions before and after functional groups modification.

2. Markov decision process configures

We extracted atoms with occurrence more than 0.01% in ChEMBL database as follows: $\{H, C, N, O, F, P, S, Cl, Br, I\}$. We internally explored the impact of the atom set composition on the performance, and found the atom set $\{C, N, O\}$ can reduce the complexity with the best performance. Other atoms (except H) can be added by the functional group modification in the last step, while H atoms are automatically added based on the implicit valence (lone-pair electrons) of other atoms in the molecule.

The probability of the transition $Pa(s, s')$ equals to 1 in this specific molecule generation task since the corresponding next molecule state s' is uniquely identified by the current molecule state s and the action a. The reward $Ra(s, s')$ is set as the objective functions consisting of experience-based metrics and QAscores obtained by the QA model. Since no single objective function will work perfectly for all the molecules, here, $Ra(s, s')$ consists of multiple objective functions derived from multiple desired properties of drugs, resulting in a multi-objective optimization problem of the computational design. One solution is to convert it to a single-objective optimization problem by a weighted summation of the multiple objective functions. However, the correlation among different objective functions is complex, and even need to dynamically change for different drugs in the design task. Thus, converting the multi-objective functions into a weighted combination of single-objective functions will result in the information loss. Thus, a more effective multi-objective optimization method is needed for the drug design task.

Suppose that an MDP process (also called as 'Episode') has a total of n steps, and the random variable 'Discount Return' U_t is defined as the total discounted reward after the time *t* (the reward before the time *t* can be ignored since it has already been observed) as follows:

$$U_t = \sum_{i=0}^{n-t} \gamma^i R_{t+i} \tag{4}$$

where γ represents the discount factor, the closer it is to 0, the more the model focuses on short-term returns.

3. The DQN algorithm pipeline

Algorithm.

Initialize the memory M with the predefined capacity N

Initialize the eval Q network with parameters ω

Initialize the target Q network with parameters $\omega' = \omega$

For episode = 1, MAX_EPISODE **do**:

  For step = 1, MAX_STEP **do**:

    Choose an action

$$a_t = \begin{cases} \arg max_{a\in A}\ Q_{eval}(s_t, a; \omega) & at\ probability\ \ 1-\varepsilon \\ random(A) & at\ probability\ \ \varepsilon \end{cases}$$

    Execute the action a_t to receive the reward r_t and the next state S_{t+1}

    Store the transition (S_t, a_t, r_t, S_{t+1}) in the memory M

    Randomly sample a minibatch transition (S_i, a_i, r_i, S_{i+1}) from the memory M

    Calculate $q\_eval = Q_{eval}(s_i, a_i; \omega)$

    Calculate

$$q\_target = \begin{cases} r_i + \gamma \cdot Q_{target}(s_{i+1}, a; \omega') & for\ \ the\ terminal\ step\ \ i+1 \\ r_i & for\ \ non\ terminal\ step\ \ i+1 \end{cases}$$

    Calculate $loss = MSEloss(q\_target, q\_eval)$

    Execute backpropagation with the $loss$

If (step % FREQUENCY == 0):

Update the target Q network parameters $\omega' = \omega$

End For

End For

4. Implementation details for QADD

In QADD, we use the Kekule formula to represent aromatic compounds, that is, aromatic bonds are treated as a combination of single and double bonds. For example, if we add a bond between carbons 1-6, 2-3, and 4-5 of a cyclohexane, the Kekule formula of the benzene will be generated. Although the Kekule formula is formally used, it does not affect the aromaticity of the atoms and bonds of the generated molecule.

The DQN in QADD consists of two Q networks with the same structure: an eval Q network and a target Q network. The two Q networks have different parameters to reduce the estimation bias of the Q value, and the loss function is defined as the MSE loss between the target Q value and eval Q value. In the Q network, molecules are converted from SMILES strings to 2048-dimensional Morgan fingerprints through RDKIT package. It consists of five fully connected layers with dimensions of 1024, 512, 128, 32, and 2.

For the multi-objective DQN configuration, an individual pair of target Q and eval Q networks are built for each reward function. And the final Q value is calculated by the average weighted summation of the Q value predicted by each eval Q network.

In the QA model, molecules are converted from SMILES strings to 'mol' format using RDKIT [41] package, and 29-D node features consist of 'Atom Symbol' (19-D), 'Atom In Ring' (2-D), 'Atom Hybridization' (6-D), 'Implicit Valence' (1-D), and 'Atom Degree' (1-D). The node features are converted into one-hot vectors. Then, DGL package [42] converts molecules into the graphs as the input of the GIN network. The network consists of 5 GIN layers and outputs the graph embeddings through a readout layer.

For the feeding back, the iteration frequency of generated molecules is set to 5000 episodes to ensure enough negative samples, where the QA model is retrained after every 5000 episodes of the RL model.

Supplementary figures

The property distributions of QED, SAscore, molecular weight, logP, and molecular topological polar surface area (TPSA) of our benchmark dataset are shown in Figure S4.

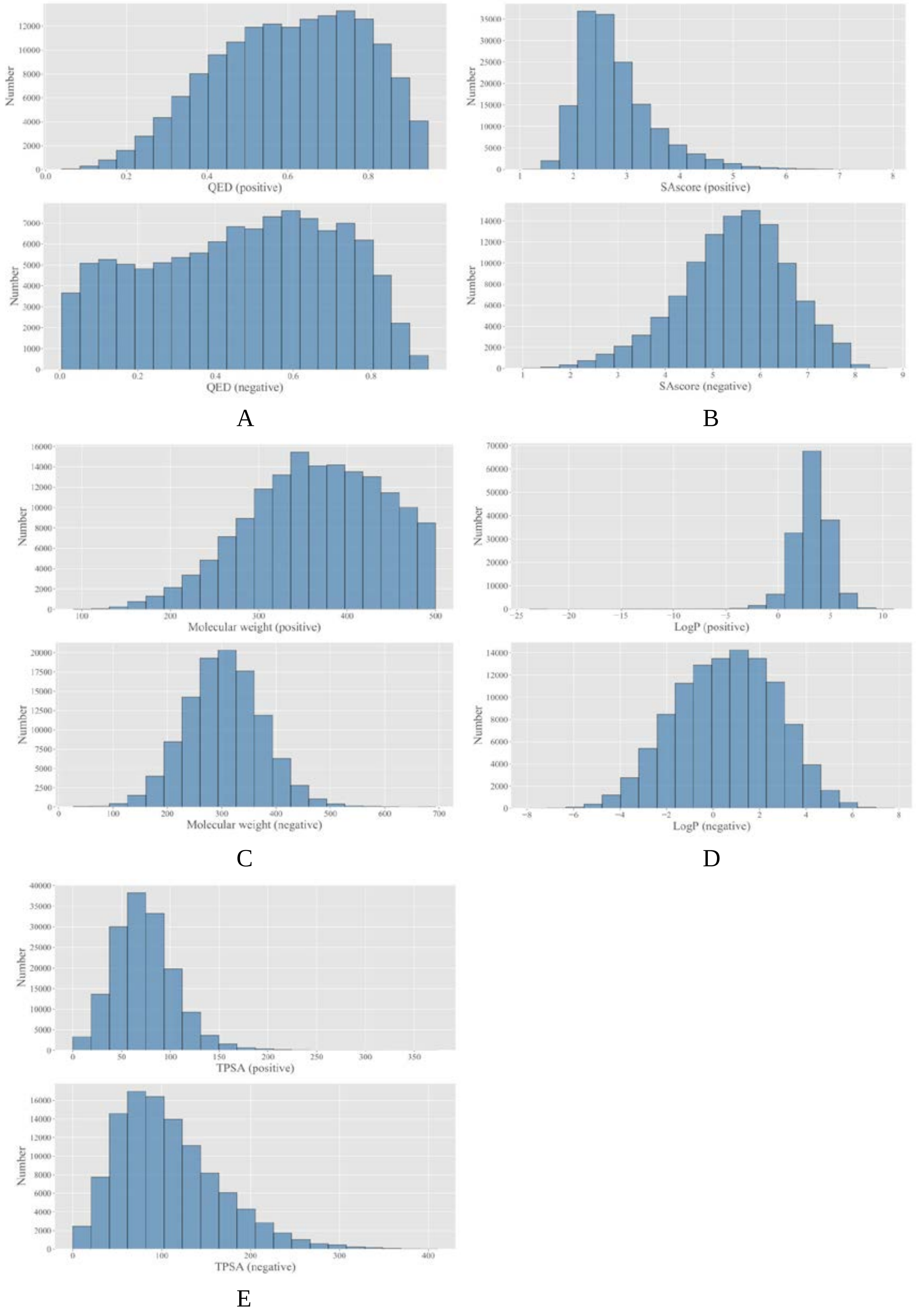

Figure S4. Property distributions of the benchmark dataset with 154,000 positive samples and 108,859 negative samples.

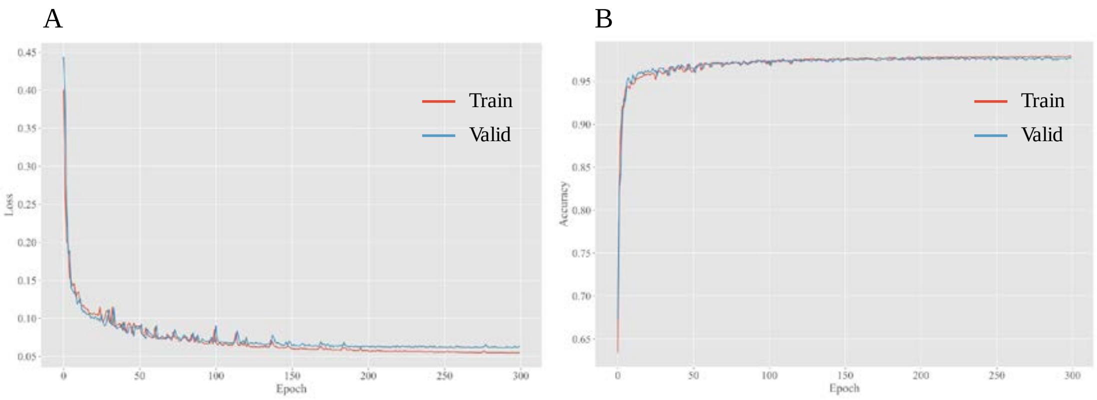

Figure S5. The accuracy (A) and loss (B) of the 3rd iteration QA model on the training set and validation set

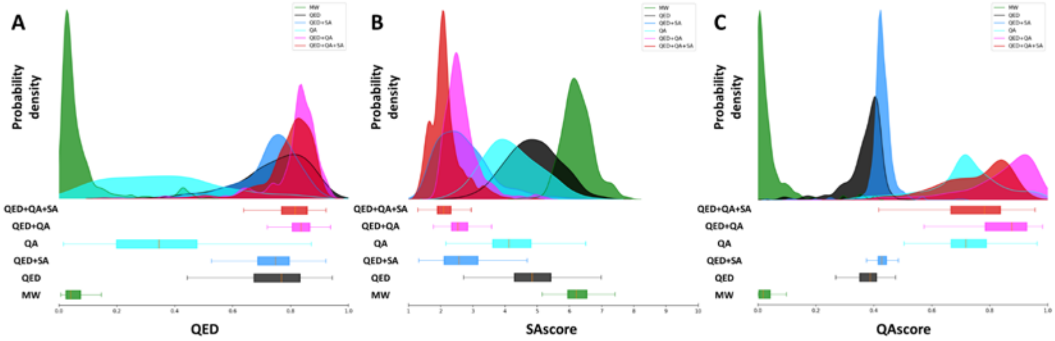

Figure S6. QED (A), SAscore (B), and QAscore (C) distributions of the generated molecules under different combinations of reward functions. 'MW' represents the molecular weight reward function; 'QED' represents the QED reward function; 'SA' represents the SAscore reward function; 'QA' represents the quality assessment reward function.

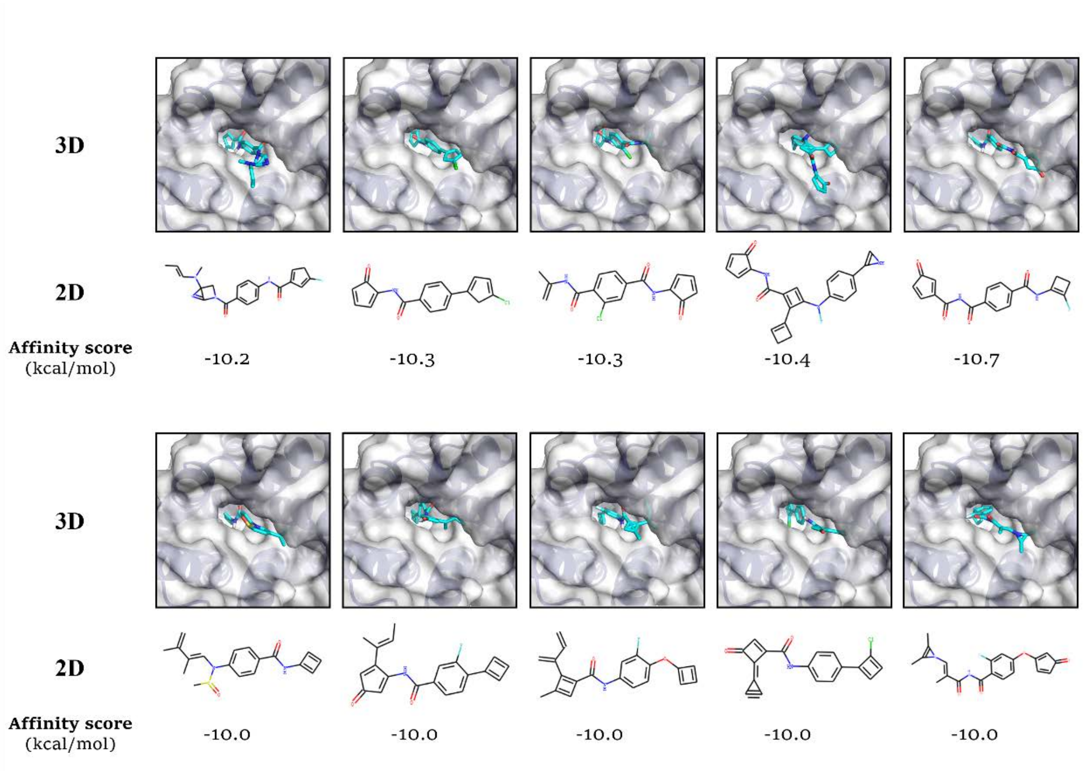

Figure S7. The docking structure of the DRD2 protein and generated molecules (blue) by QADD with top-10 predicted binding affinity.

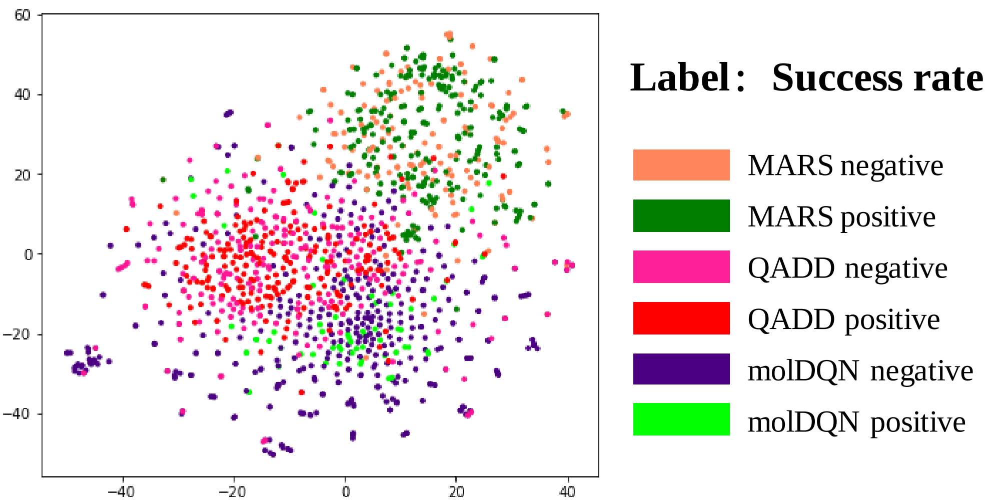

Figure S8. T-SNE visualization of molecules generated by three different methods.

Supplementary tables

Table S1. The SMILES strings of the top-10 generated molecules and 8NU.

Index (name)	SMILES string
1	CC=CN(C)C12CN(C(=O)c3ccc(NC(=O)C4=CCC(F)=C4)cc3)C1=N2
2	O=C(NC1=CC=CC1=O)c1ccc(C2=CC=C(Cl)C2)cc1
3	O=C(Nc1ccc(C2=CC=C2Cl)cc1)C1=CC(=O)C1=C1C#C1
4	C=CC(=C)C1=C(C(=O)Nc2ccc(OC3=CC=C3)c(F)c2)C=C1C
5	C=C(C)NC(=O)c1ccc(C(=O)NC2=CC=CC2=O)cc1Cl
6	CC=C(C)C1=CC(=O)C=C1NC(=O)c1ccc(C2=CC=C2)c(F)c1
7	O=C(NC1=CC=CC1=O)C1=C(C2=CCC2)C(N(F)c2ccc(C3=CN3)cc2)=C1
8	C=C(C)C(C)=CN(c1ccc(C(=O)NC2=CC=C2)cc1)S(C)=O
9	O=C(NC(=O)c1ccc(C(=O)NC2=C(F)CC2)cc1)C1=CC(=O)C=C1
10	CC(=CN1C(C)=C1C)C(=O)NC(=O)c1ccc(OC2=CC(=O)C=C2)cc1F
8NU	CC1=C(C(=O)N2CCCCC2=N1)CCN3CCC(CC3)c4c5ccc(cc5on4)F

Table S2. The evaluation metrics of the top-10 generated molecules and 8NU.

Evaluation metrics	1	2	3	4	5	6	7	8	9	10	8NU	Suggestions
QAscore	0.523	0.923	0.787	0.691	0.795	0.863	0.841	0.733	0.938	0.857	0.726	>0.5
QED	0.871	0.929	0.689	0.777	0.895	0.914	0.750	0.813	0.816	0.809	0.657	>0.605
SAscore	4.128	2.836	3.433	3.340	2.697	3.151	3.845	3.680	2.852	3.207s	2.736	<2.797

Table S3. The ADMET properties of the top-10 generated molecules and 8NU.

**ADMET features**	1	2	3	4	5	6	7	8	9	10	8NU	Suggestions
AMES	0	0	0	0	0	0	0	0	0	0	0	0
BBB	1	1	1	1	1	1	1	1	1	1	1	1
caco2	-4.586	-4.504	-4.43	-4.734	-4.508	-4.558	-5.03	-4.53	-4.862	-4.477	-4.79	> -5.15
CL	1.48	0.77	0.591	1.643	0.763	1.756	1.553	1.56	1.024	1.257	1.716	>15 mL/min/kg
CYP1A2-inhibitor	0	0	0	1	1	1	0	1	1	0	0	0
CYP1A2-substrate	1	1	1	1	1	1	0	1	1	1	1	0
CYP2C19-inhibitor	1	0	0	1	1	0	1	1	1	1	0	0
CYP2C19-	1	0	0	0	0	0	0	0	0	1	1	0

substrate												
CYP2C9- inhibitor	1	0	0	1	0	0	0	0	0	1	0	0
CYP2C9- substrate	0	0	1	0	0	0	0	0	0	1	0	0
CYP2D6- inhibitor	0	0	0	0	0	0	0	0	0	0	1	0
CYP2D6- substrate	0	0	0	0	0	0	0	1	0	0	1	0
CYP3A4- inhibitor	0	0	0	1	0	0	0	0	0	0	0	0
CYP3A4- substrate	1	0	0	1	0	0	0	1	1	0	1	0
DILI	1	1	1	1	1	1	1	1	0	1	1	0
F-20	1	1	1	1	1	1	1	1	1	1	1	1
F-30	1	1	1	1	1	1	0	1	1	1	1	1
FDAMDD	0	1	1	0	1	1	0	1	1	0	0	0
hERG	1	0	0	1	0	1	1	1	1	1	1	0
HHT	1	1	1	1	1	1	1	1	1	0	1	0
HIA	1	1	1	1	1	1	1	1	1	1	1	1
LD50	2.666	2.3	2.43	2.669	2.348	2.661	2.616	2.555	2.528	2.541	3.08	>500 mg/kg
logD	2.409	2.783	2.654	3.144	1.641	2.817	2.737	2.684	1.249	2.526	2.919	1~5
logP	2.838	3.349	2.968	4.595	2.356	3.868	3.332	3.45	1.713	2.955	3.59	0~3
logS	-4.45	-4.204	-4.122	-5.451	-3.783	-5.013	-4.843	-4.439	-3.848	-4.355	-4.867	> -4
Pgp- inhibitor	1	1	1	1	0	1	1	0	1	1	1	0
Pgp- substrate	0	0	0	0	0	0	0	0	0	0	0	0
PPB	88.104	95.033	88.169	94.242	92.328	95.494	87.196	88.709	87.631	90.703	86.577	>90
SkinSen	0	1	1	1	0	0	1	0	0	0	0	0
T	1.43	1.839	1.837	1.804	1.101	1.839	1.716	1.739	1.411	1.51	1.46	>0.5
VD	0.117	-0.174	-0.173	0.127	-0.742	-0.054	-0.004	-0.054	-0.744	-0.174	0.283	0.04-20L/kg
